# Extracting transcription factor binding sites from unaligned gene sequences with statistical models

**DOI:** 10.1186/1471-2105-9-S12-S7

**Published:** 2008-12-12

**Authors:** Chung-Chin Lu, Wei-Hao Yuan, Te-Ming Chen

**Affiliations:** 1 Department of Electrical Engineering, National Tsing Hua University, Hsinchu 30013, Taiwan; 2 Alpha Imaging Technology Corp., Jubei City, Hsinchu 302, Taiwan

## Abstract

**Background:**

Transcription factor binding sites (TFBSs) are crucial in the regulation of gene transcription. Recently, chromatin immunoprecipitation followed by cDNA microarray hybridization (ChIP-chip array) has been used to identify potential regulatory sequences, but the procedure can only map the probable protein-DNA interaction loci within 1–2 kb resolution. To find out the exact binding motifs, it is necessary to build a computational method to examine the ChIP-chip array binding sequences and search for possible motifs representing the transcription factor binding sites.

**Results:**

We developed a program to find out accurate motif sites from a set of unaligned DNA sequences in the yeast genome. Compared with MDscan, the prediction results suggest that, overall, our algorithm outperforms MDscan since the predicted motifs are more consistent with previously known specificities reported in the literature and have better prediction ranks. Our program also outperforms the constraint-less Cosmo program, especially in the elimination of false positives.

**Conclusion:**

In this study, an improved sampling algorithm is proposed to incorporate the binomial probability model to build significant initial candidate motif sets. By investigating the statistical dependence between base positions in TFBSs, the method of dependency graphs and their expanded Bayesian networks is combined. The results show that our program satisfactorily extract transcription factor binding sites from unaligned gene sequences.

## Background

Understanding transcription is central to understanding genetic regulatory mechanisms. The transcription of a gene is generally dependent on the presence of specific signals located at upstream regions of the core-promoter. These specific signals derive from their use as binding sites by transcription factors (TFs), and are therefore termed transcription factor binding sites (TFBSs). Recently, chromatin immunoprecipitation followed by cDNA microarray hybridization (ChIP-chip array) has been used to identify potential regulatory sequences, but the procedure can only map the probable protein-DNA interaction loci within 1–2 kilobases resolution [1]. To find out the exact binding motifs, it is necessary to build a computational method to examine the ChIP-chip array binding sequences and search for possible motifs representing the TFBSs (motif discovery).

There are many computational TFBS motif finding tools available [2-4]. The traditional approach for finding TFBSs is to collect and align a set of promoter sequences of co-regulated genes from either the literature or systematic experiments. Numerous computational tools, such as CONSENSUS [5], EM [6], MEME [7] and the Gibbs sampler [8], have utilized the approach to identify short DNA sequence motifs which are statistically over-represented in the promoter sequences.

Other than the alignment-based motif finding algorithms in above, many approaches have tried to extend to the use of evolutionary conservation information such as phylogenetic footprinting or the detection of combinations of binding sites (termed as cis-regulatory modules; CRMs) [2,3]. Phylogenetic footprinting methods [9-11] is an approach that seeks to identify conserved regulatory elements by comparing genomic sequences between related species. However, due to the statistical nature of the approach, e.g., a small amount of closely related species, not all transcription binding sites can be found by using phylogenetic footprinting. Hence, some algorithms have emerged to combine the alignment-based motif prediction with phylogenetic footprinting such as PhyloGibbs [12] and MY sampler [13]. On the other hand, by the detection of CRMs due to the cooperative interactions between TFs, algorithms like those in [14-16] can produce predictions of substantially better specificity than those of isolated sites.

Recently, more effective motif finders, e.g., MDscan [1], ANN-Spec [17], DMOTIFS [18], DME [19] and Cosmo [20], have taken the advantage of a background set, serving as a negative control. The goal of these discriminant motif finders is to search only for motifs that are most discriminating, that is, only those enriched in the foreground set relative to the background set [2]. Although these motif finders have improved the performance of TFBS prediction, it is still a trouble to have a satisfactory solution. How to find out accurate binding motifs may require much attention in the computational biology community. In this study, an improved sampling algorithm is proposed to incorporate the binomial probability model to build significant initial motif sets. By investigating the statistical dependence between base positions in TFBSs, it appears feasible to use statistical models to formulate the structural dependence of a motif in the identification of TFBSs. In light of this observation, the method of dependency graphs and their expanded Bayesian networks [21] is combined and prediction results show that our algorithm is able to find out motifs more consistent with previously known evidence.

## Methods

Let *TF *be one of the transcription factors to be investigated. The binding dataset of the transcription factor *TF*, denoted as *B*_*TF*_, consists of the sequences with low binding *p*-value (< 0.001) to the *TF *in the ChIP-chip array data [22]. A sliding window of size *w *is used to extract segments of length *w *when sliding through each of the sequences in *B*_*TF*_.

Let *S*_*TF *_be the collection of all extracted segments from *B*_*TF*_, *M *the number of sequences in the binding dataset *B*_*TF*_, *L*_*i *_the length of the *i*th sequence in the binding dataset *B*_*TF*_, and *T*_*TF *_the total number of segments in *S*_*TF*_. Then

$$T_{TF}=\sum_{i=1}^{M}\left(L_{i}-w+1\right).$$

To discover the binding motifs of the transcription factor *TF*, a number of initial candidate motif sets for *TF *is subsequently built from the collection *S*_*TF *_of extracted segments. Note that the contents of segments, called patterns, in *S*_*TF *_may not be distinct.

Most of early motif finding algorithms, such as Gibbs sampler [8] and MEME [23], have a weakness, where initial candidate motif sets are built by randomly extracting segments from sequences in the binding dataset *B*_*TF *_(i.e. randomly selecting segments from the set *S*_*TF*_). To improve the deficiency, the binomial probability distribution model is firstly utilized in the establishment of a number of initial candidate motif sets in our algorithm.

Then in the process of iterative sampling in our algorithm to expand and/or trim each of the initial candidate motif sets, the method of dependency graphs and their expanded Bayesian networks [21] is used to develop a statistical model for the background motif set identified as the union *S *= ∪_*TF*_*S*_*TF *_of segments extracted from all transcription factor binding datasets.

The basic procedure to find the binding motifs of the transcription factor *TF *is as follows:

1. Build *N *initial candidate motif sets.

(a) Take *N *distinct patterns from the set *S*_*TF *_with the most highest significance scores as the candidates by the binomial distribution model (see the Binomial probability distribution model subsection).

(b) Then for each of the *N *significant binding site candidates for the transcription factor *TF*, in view of evolution, collect all segments in *S*_*TF *_whose patterns have no more than *d *Hamming distance matching to the candidate pattern to form an initial candidate motif set.

At this stage, *N *initial candidate motif sets for the transcription factor *TF *are built.

2. Iteratively sample through the binding dateset *B*_*TF *_to expand and/or trim each of the *N *initial candidate motif sets so that their approximate maximum a posteriori (AMAP) scores [1,24] can keep increasing until the *N *candidate motif sets are invariant in *K *consecutive iterations (see the Iterative sampling subsection).

(a) In the calculation of AMAP scores in this stage, the background model for the background motif set *S *= ∪_*TF*_*S*_*TF *_is established under the method of dependency graphs and their expanded Bayesian networks (see the Method of dependency graphs and their expanded Bayesian networks subsection).

3. Refine each of the *N *candidate motif sets by re-examining all the segments already included in the motif set. A segment is removed from the motif set if doing so increases the AMAP score.

**Figure 1:**
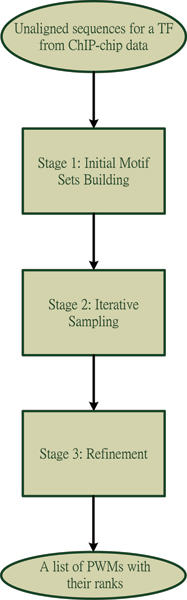
A flowchart of our algorithm.

**Figure 2:**
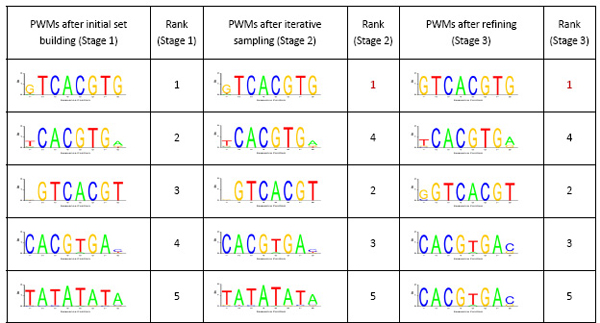
An illustration of the dynamics of the PWM and the rank of five candidate motif sets at different stages of our algorithm in the motif prediction of the transcription factor CBF1.

A simple flowchart for our algorithm is shown in Figure 1. The following subsections will expatiate on each stage of our algorithm. As an illustration of the dynamics of the PWM and the rank of different candidate motif sets at different stages of our algorithm, a summary of the prediction process for the motif of the transcription factor CBF1 is given in Figure 2.

### Initial motif sets building

Our method begins by enumerating those patterns in *S*_*TF *_that appear most often in the binding dataset *B*_*TF *_than in others. What we want to do first is to calculate the appearance probability of a pattern in *S*_*TF*_, which is the probability that the pattern appears no less than *n *times in the binding dataset *B*_*TF*_. If a pattern *b *appears more often than other patterns in *S*_*TF *_and its occurrence probability in a generic intergenic region is comparatively low, the calculated significance score of *b *would be relatively high. We will take patterns with the most highest significance scores as the candidates to build a number of initial candidate motif sets.

#### Binomial probability distribution model

The probability to observe exactly *j* occurrences of pattern *b *in the collection *S*_*TF *_of segments extracted from the binding dataset *B*_*TF *_is estimated by the binomial distribution

$$P_{TF}(occ(b)=j)=\left(\begin{array}{c} T_{TF}\\ j \end{array}\right)\times {\left(f(b)\right)}^{j}\times {\left(1-f(b)\right)}^{T_{TF}-j},$$

where *occ*(*b*) is the occurrence times of pattern *b *in *S*_*TF *_and *f *(*b*) is the probability that pattern *b *occurs in the intergenic region and is estimated as the relative frequency of pattern *b *in the union *S *= ∪_*TF*_*S*_*TF *_of segments extracted from all transcription factor binding datasets. The probability to observe *n *or more occurrences of the pattern *b *in *S*_*TF *_is

(1) $$P_{TF}(occ(b)\geq n)=1-\sum_{j=0}^{n-1}P_{TF}(occ(b)=j).$$

We define the significance score *sig*_*TF *_(*b*) of a pattern *b *to *TF *as

*sig*_*TF *_(*b*) = -log_10_(*P*_*TF *_(*occ*(*b*) ≥ *n*)).

The less probable pattern *b *in *S *appears more than *n *times in *S*_*TF*_, the more probable will it be a binding site candidate for the transcription factor *TF*. We will take *N *distinct patterns with the most highest significance scores as the candidates.

For each of the *N *significant binding site candidates for the transcription factor *TF*, in view of evolution, collect all segments in *S*_*TF *_whose patterns have no more than *d *Hamming distance matching to the candidate pattern to form an initial candidate motif set. Thus *N *initial candidate motif sets for the transcription factor *TF *are built at the end of this stage. As an example, the PWM and the rank of five initial candidate motif sets for the motif prediction of the transcription factor CBF1 are shown in Figure 2.

### Iterative sampling

In this stage, a sampling method is used to expand and/or trim each of the *N *initial candidate motif sets *M*_1_, *M*_2_,.., *M*_*N*_. For our purpose, a false motif set *M*_*N*+1 _is created by randomly selecting *e*_0 _(*e*_0 _is equal to the maximum size of the *N *initial candidate motif sets) segments from the collection *S*_*TF *_such that *M*_*i *_∩ *M*_*N*+1 _= ∅, for all *i *= 1, 2,..., *N*. In addition, let the collection *S *= ∪_*TF*_*S*_*TF *_of segments extracted from all transcription factor binding datasets represent the intergenic background and here be denoted as *M*_*BG*_.

#### Approximate maximum a posteriori (AMAP) measure

The score $amap_{M_{i}}$ of the approximate maximum a posteriori (AMAP) measure of the candidate motif set *M*_*i *_is defined as [1,24]

$$amap_{M_{i}}=\frac{1}{w}\left\{\sum_{s=0}^{w-1}\sum_{j\in A,T,G,C}p_{s,j}\log(p_{s,j})-\frac{1}{n_{i}}\sum_{m\in M_{i}}\log(P(m|M_{BG}))\right\},$$

where *p*_*s*, *j *_is the frequency of nucleotide *j *at base position *s *in the candidate motif set *M*_*i*_(which can be retrieved from the position specific scoring matrix (PSSM) of *M*_*i*_), *n*_*i *_is the number of segments in *M*_*i*_, and *P*(*m*|*M*_*BG*_) is the probability of the pattern of segment *m *in the motif set *M*_*i *_under an expanded Bayesian network (EBN) model [21] developed from the background motif set *M*_*BG *_(EBN model will be discussed shortly).

The first part of the AMAP score is a negative entropy, which is higher if there are more similar patterns in the candidate motif set *M*_*i*_. A motif set *M*_*i *_with all identical patterns has the maximum negative entropy 0, whereas equal nucleotide frequencies at every position in the PSSM of *M*_*i *_has the minimum negative entropy. And a segment *m *in the candidate motif set *M*_*i *_which has a pattern much different from the background motif model built from *M*_*BG *_would have lower appearance probability *P *(*m*|*M*_*BG*_) and hence increases the score $amap_{M_{i}}$ of the AMAP measure of *M*_*i*_.

#### Sampling strategy

In each iteration, there are two steps for the sampler, the S-step and the M-step.

In the S-step, the sampler samples a site by randomly selecting a sequence from *B*_*TF *_and then randomly picking up a site in the selected sequence to extract a segment *m*_*s *_of length *w*. For 1 ≤ *i *≤ *N*, if the sampled segment *m*_*s *_appears in *M*_*i*_, segment *m*_*s *_will be removed from *M*_*i *_if the AMAP score $amap_{M_{i}}$ of the candidate motif set *M*_*i *_increases after its removal; otherwise, segment *m*_*s *_will be kept in *M*_*i*_. Note that the PSSM of the motif model *M*_*i *_should be retrained if the sampled segment *m*_*s *_is removed from *M*_*i*_.

Which one of the *N *+ 1 motif sets would be the best motif set for the sampled segment *m*_*s *_will depend on the appendant score $app_{M_{i}}$ that the segment *m*_*s *_is derived from *M*_*i *_[24,25]

(2) $$app_{M_{i}}=\log\left(\frac{P(n_{i})}{1-P(n_{i})}\frac{P(m_{s}|M_{i})}{P(m_{s}|M_{BG})}\right),1\leq i\leq N+1,$$

where *n*_*i *_is the size of current motif set *M*_*i*_, *P*(*n*_*i*_) equals $\frac{n_{i}}{T_{TF}}$, *P*(*m*_*s*_|*M*_*i*_) and *P*(*m*_*s*_|*M*_*BG*_) are the probabilities of the content of the sampled segment *m*_*s *_under the PSSM model developed from the current motif set *M*_*i *_and under an EBN model developed from the background *M*_*BG*_, respectively. The sampled segment *m*_*s *_will be considered to append into the motif set *M*_*i *_with the highest appendant score $app_{M_{i}}$. If $app_{M_{N+1}}$ is the highest score, then the sampled segment *m*_*s *_is appended into the false motif set *M*_*N*+1 _unless *m*_*s *_is already there and the current iteration stops here. If for some *i*, 1 ≤ *i *≤ *N*, $app_{M_{i}}$ is the highest score, the sampled segment *m*_*s *_will be further checked in the M-step to see if we really want to append *m*_*s *_into *M*_*i *_unless we have processed *m*_*s *_for *M*_*i *_at the beginning of this S-step as in above and the current iteration stops here.

In the M-step, the sampler has to decide whether the newly sampled segment *m*_*s *_should be appended into the candidate motif set *M*_*i *_or not. The AMAP measure again will be used to evaluate our decision. The sampled segment *m*_*s *_is appended into the candidate motif set *M*_*i *_if and only if the score $amap_{M_{i}}$ of the motif model *M*_*i *_is increased once the sampled segment *m*_*s *_is appended to *M*_*i*_. Note that the PSSM of the motif model *M*_*i *_should be retrained after the sampled segment *b*_*s *_is appended to *M*_*i*_. Now the M-step is done and the current iteration stops here.

The sampler will iteratively sample through the binding dataset *B*_*TF *_to expand and/or trim the *N *candidate motif sets *M*_1_, *M*_2_,.., *M*_*N *_so that their AMAP scores $amap_{M_{i}}$ will keep increasing. The *N *candidate motif sets will tend to be invariant after a (larger) number of iterations. The stopping criterion of the sampling process is that all the *N *candidate motif sets are invariant in *K *consecutive iterations. The parameter *K *is usually set to be 1% of the size of *S*_*TF*_.

#### Alternative sampling strategy

There is an alternative sampling strategy as follows.

In the S-step, the new sampler also randomly samples a site from a sequence in *B*_*TF *_to extract a segment *m*_*s*_of length *w*. For 1 ≤ *i *≤ *N*, if the pattern of the sampled segment *m*_*s *_appears in *M*_*i*_, all the segments in *M*_*i *_whose pattern is the same as that of *m*_*s *_will be removed if the AMAP score $amap_{M_{i}}$ of the motif set *M*_*i *_increases after their removal. Otherwise, these segments will be kept in *M*_*i*_.

Also in the S-step, if $app_{M_{N+1}}$ is the highest among all $app_{M_{i}}$, 1 ≤ *i *≤ *N *+ 1, then all segments in the set *S*_*TF *_having the same pattern as that of the sampled segment *m*_*s *_will be appended into the false motif set *M*_*N*+1 _unless these segments are already there and the current iteration stops here. If $app_{M_{i}}$ is the highest for some *i*, 1 ≤ *i *≤ *N*, the sampled segment *m*_*s *_will be further checked in the M-step to see if we really want to append those segments in the set *S*_*TF *_having the same pattern as that of the sampled segment *m*_*s *_into *M*_*i *_unless we have already processed those segments for *M*_*i *_at the beginning of this S-step as in above and the current iteration stops here.

In the M-step, all the segments in the set *S*_*TF *_having the same pattern as that of the sampled segment *m*_*s *_are decided to append to the candidate motif set *M*_*i *_if and only if the AMAP score $amap_{M_{i}}$ of *M*_*i *_increases after these segaments are appended into *M*_*i*_.

#### Method of dependency graphs and their expanded Bayesian networks

Considering the binding mechanism of transcription factors to specific DNA sites (motifs), there must be distinctive features for the specific motif regions from other intergenic regions which represent the background DNA sequence. Hence, it is conceivable that we can use a statistical model to capture the feature of a specific DAN site (motif) or a generic DNA intergenic region (background). Since the size of a candidate motif set *M*_*i *_is often small, a PSSM model is commonly used for *M*_*i *_instead of any other more sophisticated statistical model. However, the size of the background motif set *M*_*BG *_is usually large enough to be equipped with a more sophisticated one.

**Figure 3:**
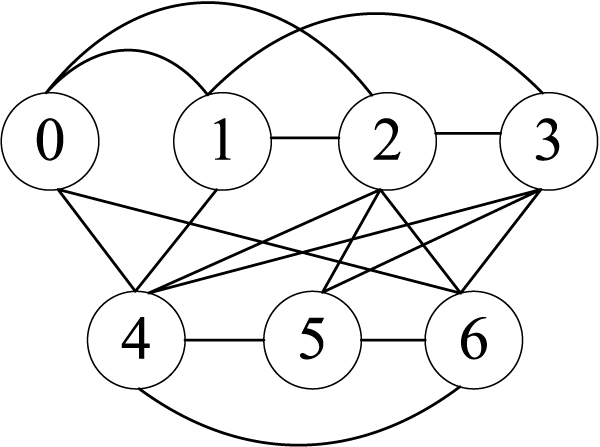
An example of dependency graph.

As reported in [21], a dependency graph model is used to fully capture the intrinsic interdependency between base positions in a motif or region. The establishment of dependency between two positions is based on a *χ*^2^-test from known sample data. An edge is established between two nodes (a node represents a base position) in the graph if the two corresponding base positions of the motif or region are dependent. After all dependent edges have being established completely, a dependency graph for the motif or region is constructed. An example of a dependency graph with 7 nodes is shown in Figure 3.

**Figure 4:**
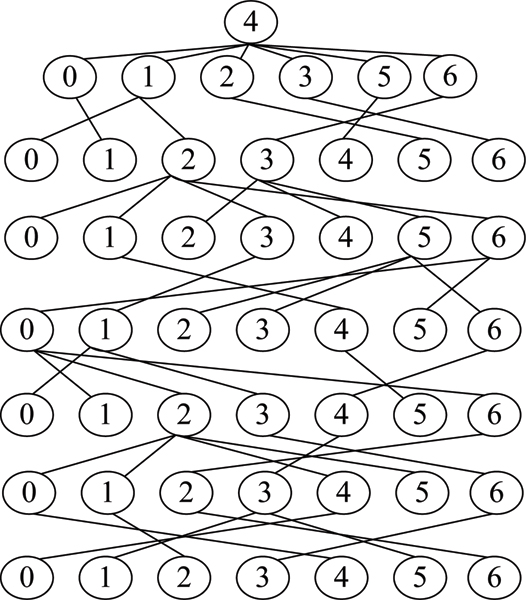
An example of expanded Bayesian network.

As reported in [21], although the dependency graph can fully capture the intrinsic interdependency between base positions in a motif or region, it is difficult, if not impossible, to perform statistical inference based on the dependency graph. To resolve the dilemma, the dependency graph is expanded to form a Bayesian network (which is a directed acyclic graph that facilitates statistical reasoning) by allowing a base position in the dependency graph to appear more than once in the Bayesian network as nominally distinct nodes. Figure 4 shows an example of an expanded Bayesian network of the dependency graph in Figure 3. For the detailed procedure of constructing an expanded Bayesian network (EBN) from a dependency graph, please see [21]. In this paper, we use EBNs to model the background motif set *M*_*BG*_.

Continued with the same example of the motif prediction of the transcription factor CBF1, the PWM and the rank of the five candidate motif sets at the end of the iterative sampling stage are also shown in Figure 2, together with the final results at the end of the refinement stage.

## Results and discussion

### Data

In order to search for the transcription factor binding sites that regulate gene expressions, we collected binding promotor sequences from the cDNA microarray hybridization (ChIp-chip array) of yeast genome [22]. Each of the binding sequences may contain some unknown motifs that are implanted at unknown positions. These data represent the binding affinity of a target transcription factor to the promoter region of a gene *in vivo*. The experiment protocol assigns a binding *p*-value to each binding promoter sequence of the corresponding transcription factor. A sequence with binding *p*-value less than 0.001 is considered to be bound by the corresponding transcription factor. The threshold of 0.001 is set up to reduce the false positive identification in yeast genome-wide screening.

We collected the ChIp-chip array sequence data from the "Motif discovery results – Discovered motifs, version 24" at [26]. For a transcription factor *TF *to be investigated, we collected all sequences with binding *p*-value less than the threshold 0.001 to *TF *into the binding dataset *B*_*TF*_. There are 65 binding datasets *B*_*TF *_being able to be collected from Harbison's website.

### Accuracy measurement and comparison

To evaluate the performance of our program, we collected known specificities from many famous websites, such as YPD, SCPD, Transfac and from the literature with experimental evidence [27] to compare with the discovered specificities predicted by our program.

**Figure 5:**
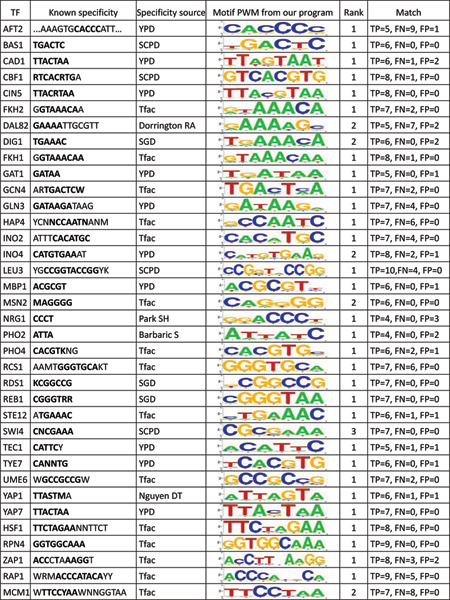
**Predicted results of our program compared with known evidence**. The letter symbols used in the 'Known specificity' column have the following mapping: aA: a tT: t gG: g cC: c wW: at rR: ag mM: ac kK: tg yY: tc sS: gc dD: atg hH: atc vV: agc bB: tgc nN: atgc

Among the 65 binding datasets *B*_*TF *_collected from Harbison's website, we chose 36 transcription factor binding datasets which have known specificities with experimental evidence to evaluate the performance of our program. The results of our program for the 36 transcription factor binding datasets are listed in Figure 5. It is deserved to be mentioned that the specificity reported for transcription factor PHO2 in Harbrison *et al*'s website is "GTGCGsyGCG", while the predicted result of our program is "ATTATC". In this case, the newly found motif by our program is more consistent with the results reported by Barbaric *et al *[28] that PHO2 binds to an AT-rich region than the specificity reported in Harbrison *et al*'s website.

In this study, we compared our program with two online programs, MDscan [29] and Cosmo [30]. MDscan is a famous program that can be used to examine the ChIP-array selected sequences and search for DNA sequence motifs representing the protein-DNA interaction sites. It takes the advantage of combining two widely adopted motif search strategies, word enumeration and position-specific weight matrix updating, and incorporates the ChIP enrichment information to accelerate the search and enhance its success rate. The comparison of MDscan with our program is shown in Table 1. Also reported in Table 1 is the performance of our algorithm when the the PSSM model [8] instead of the EBN model [21] is used to model the background motif set *M*_*BG *_in the calculation of the AMAP scores of the *N *candidate motif sets and the appendant scores of the *N *+ 1 motif sets. In Table 1, for each transcription factor, the number in each 'Rank' column indicates the rank of the predicted motif which is most consistent with the known evidence from the top ten predicted candidate motifs.

**Table 1:** Comparison of MDscan and our program.

TF	Rank (EBN model)	Rank (PSSM model)	Rank (MDscan)
AFT2	1	1	1
BAS1	1	5	2
CAD1	1	5	4
CBF1	1	2	1
CIN5	1	2	1
FKH2	1	1	3
DAL82	2	1	N*
DIG1	2	3	1
FKH1	1	2	1
GAT1	1	2	2
GCN4	1	1	1
RPN4	1	1	1
GLN3	1	1	1
HAP4	1	3	1
INO2	1	2	1
INO4	2	2	1
LEU3	1	1	1
MBP1	1	1	2
MSN2	3	3	4
NRG1	1	2	1
PHO2	1	1	2
PHO4	1	2	1
RCS1	1	1	5
RAP1	1	3	2
RDS1	1	1	1
REB1	1	3	4
STE12	1	2	1
SWI4	3	2	4
TEC1	1	4	1
TYE7	1	1	4
UME6	1	1	3
YAP1	1	5	1
YAP7	1	2	1
HSF1	1	1	1
AZF1	1	1	N
MCM1	2	2	N

* N means that the program predicts no motif.

As shown in Table 1, our approach with EBN background model outperforms the other two methods. Our approach with EBN background model gives 30 out of the 36 most predicted motifs for the corresponding 36 transcription factors with the 1st rank, while MDscan and our approach with PSSM background model give only 20 out of 36 and 15 out of 36 most predicted motifs with the 1st rank, respectively. Moreover, MDscan fails in discovering a motif for three transcription factor binding datasets, while our approach in this study is still able to predict a motif consistent with the known evidence.

Cosmo (constrained search for motifs) is a general purpose algorithm for conserved motif detection that allows the search to be supervised by specifying a set of constraints that the PWM of the unknown motif must satisfy. Such constraints may be formulated derived from prior biological knowledge about the structure of the transcription factor, such as the length of the motif intervals. Although Cosmo is based on the same two-component multinomial mixture model used in MEME, it employs the likelihood principle instead of the *E*-value criterion in MEME. In addition, three model types (OOPS, ZOOPS, or TCM) can data-adaptively be selected in Cosmo to achieve better performance. Since there is no prior knowledge used in our program, we compared it to the constraint-less version of the Cosmo program. On the other hand, since the Cosmo program reports only one motif PWM for a dataset, instead of a list of ranked candidate motif PWMs as in MDscan, we adopted only the rank 1 results of our program in this comparison. To evaluate the performance of both programs, we used the statistics proposed by Tompa et al. [4]. For a (computational) tool at the site level, the performance statistics on a dataset are defined as follows:

$$\begin{array}{ccc} \begin{array}{r} \text{Sensitivity}\\ \text{Positive predictive value}\\ \text{Average site performance} \end{array} & \begin{array}{c} :\\ :\\ : \end{array} & \begin{array}{c} Sn=TP/(TP+FN)\\ PPV=TP/(TP+FP)\\ ASP=(Sn+PPV)/2 \end{array} \end{array}$$

where *TP *is the number of known sites overlapped by predicted sites, *FN *is the number of known sites not overlapped by predicted sites, and *FP *is the number of predicted sites not overlapped by known sites. To summarize the performance of a given tool over a collection C of datasets, we compute the "combined" statistics as though *C *were one large dataset by adding *TP*, *FP *and *FN *respectively over the datasets in C. Then the combined statistics of our program are *Sn *= 0.6698, *PPV *= 0.8206, and *ASP *= 0.7452, while those of Cosmo are *Sn *= 0.6573, *PPV *= 0.5134 and *ASP *= 0.5854. For the detailed Cosmo prediction results and the comparison of the two programs, please see Figure S1 (see Additional file 1). The comparison shows that our program can offer better performance than Cosmo, especially in the elimination of false positives.

The parameters used in our program include the sliding window size *w *used to extract segments from binding datasets *B*_*TF *_to form *S*_*TF*_, the Hamming distance *d *used to collect segments from *S*_*TF *_to establish initial candidate motif sets, the number of most dependent edges used to form a dependency graph for the background motif model and the number of parents used in the construction of an expanded Bayesian network from the dependency graph [21]. The parameters used in our program to give the best predicted motifs for each of the 36 transcription factors are listed in Table S1 (see Additional file 2). Comparing the performance of the two sampling strategies discussed in the Method section, as shown in Figure S2 (see Additional file 3), we found that the alternative sampler is faster and has almost identical best predicted motifs with those by the primary sampler, except that transcription factors GCN4, HAP4 and PHO4 have the best predicted motifs one nucleotide position shift from those by the primary sampler. In addition, the alternative sampler is slightly better than the primary sampler in the sense that the best predicted motif for the transcription factor DIG1 promotes its rank from the 2nd place by the primary sampler to the 1st place by the alternative sampler.

## Conclusion

In this study, we employed the binomial probability model to establish a number of initial candidate motif sets, and used the method of dependence graphs and their expanded Bayesian networks to model the background motif set as a control to predict TFBSs (motifs) from a set of unaligned DNA sequences. The prediction results suggest that, overall, our algorithm outperforms MDscan since the predicted motifs are more consistent with previously known specificities reported in the literature and have better prediction ranks. And when compared with the constraint-less Cosmo program, our algorithm has a slightly higher combined sensitivity *Sn*, a much higher positive predictive value *PPV *and a higher average site performance *ASP*. However, the performance of our algorithm is not much better if the length of possible binding sites are too long (more than 12 bps). Further research is needed to discover long motifs.

Furthermore, variable spacing within binding sites is legitimate for some transcription factors while this study focuses on ungapped motif discovery. Programs such as BIPAD [31] and spaced dyad [32] have investigated into such a bipartitie sequence element discovery problem. Therefore another direction for our future research is to investigate into gapped motifs.

## Competing interests

The authors declare that they have no competing interests.

## Authors' contributions

CL and WY developed and implemented the method. All authors participated in discussions and writing of the paper.

## Supplementary Material

Additional file 1 Figure S1 – Predicted results of the constraint-less Cosmo program and the comparison with our program. Click here for file

Additional file 2 Table S1 – Parameters used in our program to give the best predicted motifs for the 36 transcription factors. Click here for file

Additional file 3 Figure S2 – Comparison of the predicted results with the primary and alternative samplers. Click here for file
