# Supplementary material for: Extracting transcription factor binding sites from unaligned gene sequences with statistical models
Source: BMC Bioinformatics. 2008 Dec 12;9(Suppl 12):S7. doi: 10.1186/1471-2105-9-S12-S7 (PMC2638147; doi:10.1186/1471-2105-9-S12-S7)
Supplement: Additional file 1 — Figure S1 – Predicted results of the constraint-less Cosmo program and the comparison with our program. [file 1471-2105-9-S12-S7-S1.pdf]

Figure S1 - Predicted results of the constraint-less Cosmo program and the comparison with our program, where R.C. means "reverse complementary".

TF	Specificity	Motif PWM from the constraint-less Cosmo program	Cosmo program	Our program (rank 1 results only)
AFT2	...AAAGTGCACCC **ATT**...	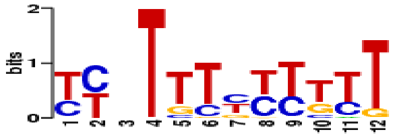	TP=2<br>FN=12<br>FP=10	TP=5<br>FN=9<br>FP=1
BAS1	TGA**CT**C	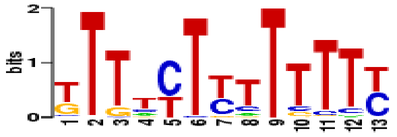	TP=2<br>FN=4<br>FP=11	TP=6<br>FN=0<br>FP=0
CAD1	TTACTAA	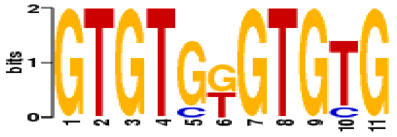	TP=0<br>FN=7<br>FP=11	TP=6<br>FN=1<br>FP=2
CBF1	RT**CACRTGA**	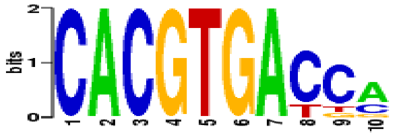	TP=7<br>FN=2<br>FP=3	TP=8<br>FN=1<br>FP=0
CIN5	**TTACRTAA**	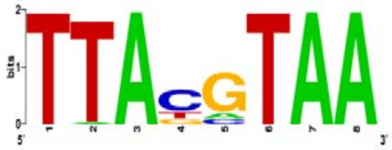	TP=8<br>FN=0<br>FP=0	TP=8<br>FN=0<br>FP=0
FKH2	G**GTAAACAA**	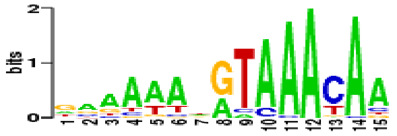	TP=8<br>FN=1<br>FP=7	TP=7<br>FN=2<br>FP=0
DAL82	**GAAA**ATTGCGTT	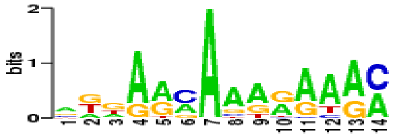	TP=4<br>FN=8<br>FP=10	TP=4<br>FN=8<br>FP=3
DIG1	**TGAAAC**	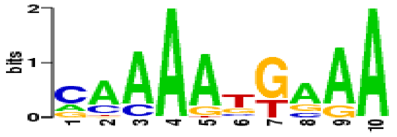	TP=5<br>FN=1<br>FP=5	TP=3<br>FN=3<br>FP=4

FKH1	G**GTAAACAA**	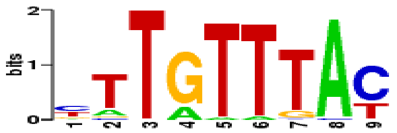	TP=8<br>FN=1<br>FP=1<br>R.C.	TP=8<br>FN=1<br>FP=0
GAT1	G**ATAA**	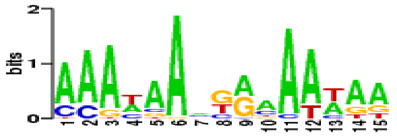	TP=4<br>FN=1<br>FP=2	TP=5<br>FN=0<br>FP=1
GCN4	AR**TGA**C**TCW**	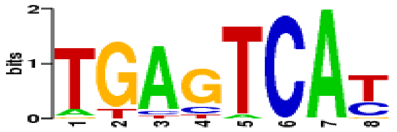	TP=6<br>FN=3<br>FP=2	TP=7<br>FN=2<br>FP=0
GLN3	GAT**AAG**ATAAG	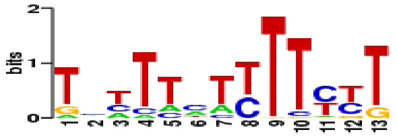	TP=3<br>FN=8<br>FP=10	TP=7<br>FN=4<br>FP=0
HAP4	**YCNNCCAATNAN**<br>**M**	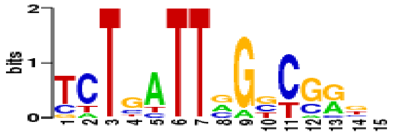	TP=13<br>FN=0<br>FP=2<br>R.C.	TP=7<br>FN=6<br>FP=0
INO2	A**TTT**CACATGC	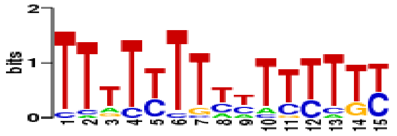	TP=3<br>FN=8<br>FP=12	TP=7<br>FN=4<br>FP=0
INO4	CATGTG**AAA**T	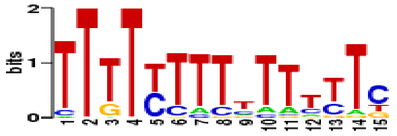	TP=3<br>FN=7<br>FP=12	TP=3<br>FN=7<br>FP=4
LEU3	YG**CCGGTACCGGY**<br>K	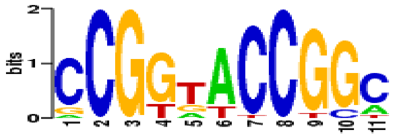	TP=11<br>FN=3<br>FP=0	TP=10<br>FN=4<br>FP=0
MBP1	**AC**G**C**GT	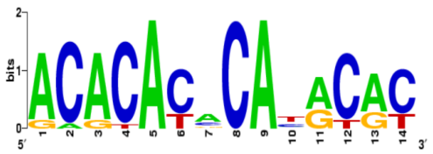	TP=3<br>FN=3<br>FP=11	TP=6<br>FN=0<br>FP=1
MSN2	**MA**GGGG	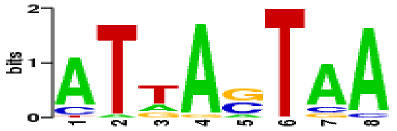	TP=2<br>FN=4<br>FP=6	TP=2<br>FN=4<br>FP=5

NRG1	CCCT	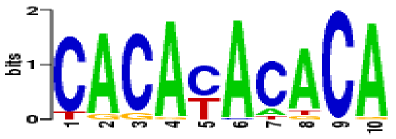	TP=0<br>FN=4<br>FP=10	TP=4<br>FN=0<br>FP=3
PHO2	A**TTA**	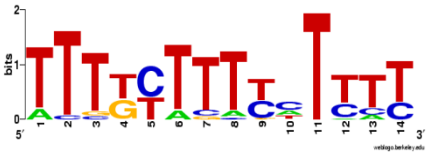	TP=2<br>FN=2<br>FP=12	TP=4<br>FN=0<br>FP=2
PHO4	CAC**GTKNG**	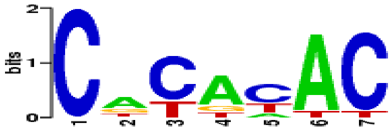	TP=5<br>FN=3<br>FP=2<br>R.C.	TP=6<br>FN=2<br>FP=1
RCS1	**AAM**TGGGTGCAK<br>T	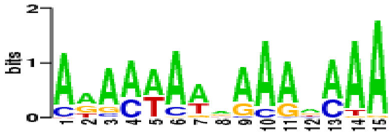	TP=3<br>FN=10<br>FP=12	TP=7<br>FN=6<br>FP=0
RDS1	K**CGGCCG**	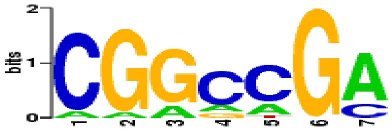	TP=6<br>FN=1<br>FP=1	TP=7<br>FN=0<br>FP=0
REB1	**CGGGTRR**	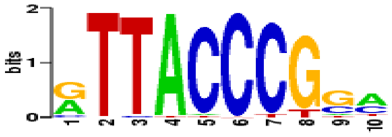	TP=7<br>FN=0<br>FP=3<br>R.C.	TP=7<br>FN=0<br>FP=0
STE12	**ATGAAA**C	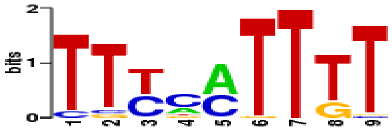	TP=6<br>FN=1<br>FP=3<br>R.C.	TP=6<br>FN=1<br>FP=1
SWI4	CNCG**AAA**	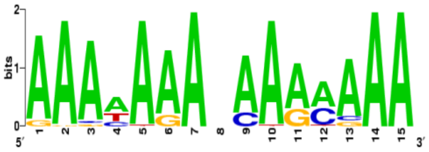	TP=3<br>FN=4<br>FP=12	TP=0<br>FN=7<br>FP=7
TEC1	CA**TTC**Y	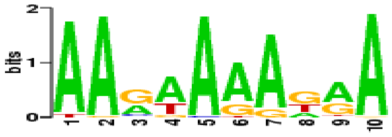	TP=3<br>FN=3<br>FP=7<br>R.C.	TP=5<br>FN=1<br>FP=1
TYE7	**CANNTG**	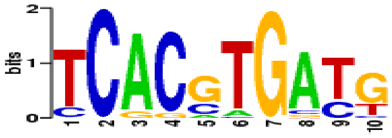	TP=6<br>FN=0<br>FP=4	TP=6<br>FN=0<br>FP=1

UME6	**WGCCGCCGW**	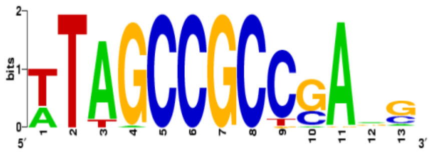	TP=9<br>FN=0<br>FP=1	TP=7<br>FN=2<br>FP=0
YAP1	**TTASTMA**	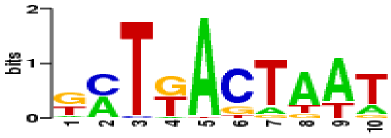	TP=7<br>FN=0<br>FP=3<br>R.C.	TP=6<br>FN=1<br>FP=1
YAP7	**TTACTAA**	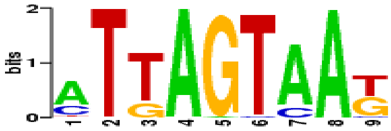	TP=7<br>FN=0<br>FP=2<br>R.C.	TP=7<br>FN=0<br>FP=0
HSF1	**TTCTAGAANNTTC T**	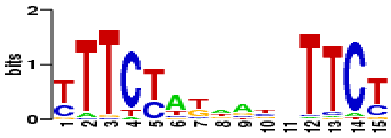	TP=13<br>FN=1<br>FP=2	TP=8<br>FN=6<br>FP=0
RPN4	GGT**GGCAA**A	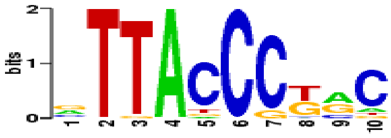	TP=4<br>FN=5<br>FP=6	TP=9<br>FN=0<br>FP=0
ZAP1	**ACCCTAAAGGT**	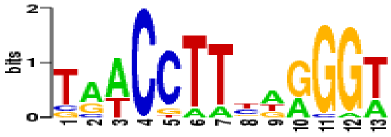	TP=11<br>FN=0<br>FP=2<br>R.C.	TP=8<br>FN=3<br>FP=2
RAP1	**WRMACCCATACA YY**	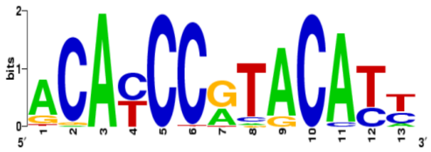	TP=14<br>FN=0<br>FP=1	TP=9<br>FN=5<br>FP=0
MCM1	**WTTCCYAAWNN GGTAA**	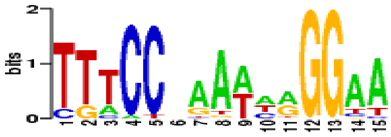	TP=13<br>FN=3<br>FP=2	TP=0<br>FN=16<br>FP=7
