# Supplementary material for: Extracting transcription factor binding sites from unaligned gene sequences with statistical models
Source: BMC Bioinformatics. 2008 Dec 12;9(Suppl 12):S7. doi: 10.1186/1471-2105-9-S12-S7 (PMC2638147; doi:10.1186/1471-2105-9-S12-S7)
Supplement: Additional file 2 — Table S1 – Parameters used in our program to give the best predicted motifs for the 36 transcription factors. [file 1471-2105-9-S12-S7-S2.pdf]

Table S1 - Parameters used in our program to give the best predicted motifs for the 36 transcription factors.

TF	Final rank	w	d	Parents	Edges	Rank of sig_{TF}
AFT2	1	6	1	1	12-4	3
BAS1	1	6	1	1	12-9	4
CAD1	1	8	1	1	2-1	4
CBF1	1	8	1	1	7-1	2
CIN5	1	8	2	1	8-1	1
FKH2	1	7	1	1	16-15 6-3	4
DAL82	2	7				5
DIG1	2	7				4
FKH1	1	8	1	1	22-17	1
GAT1	1	6	1	1	14-8	1
GCN4	1	7	1	1	2-1	3
RPN4	1	9	2	2	1	5
GLN3	1	7	1	1	15-4	1
HAP4	1	7	1	1	8-1	5
INO2	1	7	1	1	18-9	3
INO4	2	10				5
LEU3	1	10	1	2	45-1	1
MBP1	1	7	1	1	8-1	1
MSN2	3	6				4
NRG1	1	7	1	1	5-1	3
PHO2	1	6	1	1	6-4	4
PHO4	1	7	1	1	15-9	1
RCS1	1	7	1	1	2-1	4
RDS1	1	7	1	1	19-2	3
REB1	1	7	1	1	13-12	2
STE12	1	7	1	1	9-1	5
SWI4	2	7				2
TEC1	1	6	1	1	14-9	5
TYE7	1	7	1	1	7-2	1
UME6	1	7	1	1	2-1	5
YAP1	1	7	1	1	11-2	1
YAP7	1	7	2	1	6-3 1	2
HSF1	1	8	1	1	5-1	2
ZAP1	1	10	1	2	44-9 5-1	3
RAP1	1	9	1	2	15-5	5
MCM1	3	7				1
