# Supplementary material for: Extracting transcription factor binding sites from unaligned gene sequences with statistical models
Source: BMC Bioinformatics. 2008 Dec 12;9(Suppl 12):S7. doi: 10.1186/1471-2105-9-S12-S7 (PMC2638147; doi:10.1186/1471-2105-9-S12-S7)
Supplement: Additional file 3 — Figure S2 – Comparison of the predicted results with the primary and alternative samplers. [file 1471-2105-9-S12-S7-S3.pdf]

Figure S2 - Comparison of the predicted results between the primary and alternative samplers.

TF	Known specificity	Results with the primary sampler	Results with the alternative sampler	Rank
AFT2	...AAAGTG**CACCC**ATT...	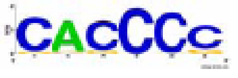	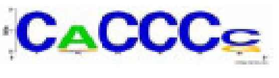	1
BAS1	**TGACTC**	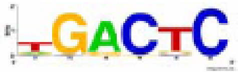	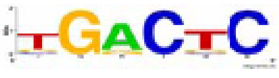	1
CAD1	**TTACTAA**	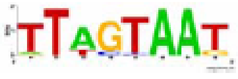	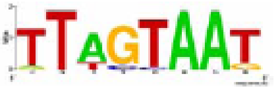	1
CBF1	**RTCACRTG**A	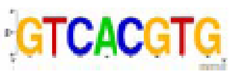	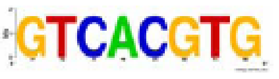	1
CIN5	**TTACRTAA**	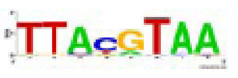	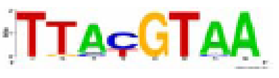	1
FKH2	G**GTAAACA**A	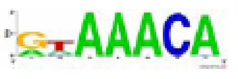	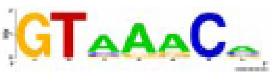	1
DAL82	**GAAAA**TTGCGTT	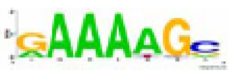	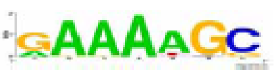	2
DIG1	**TGAAAC**	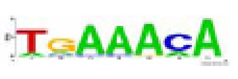	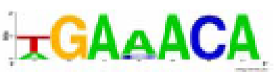	2→1
FKH1	G**GTAAACAA**	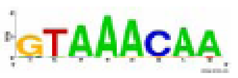	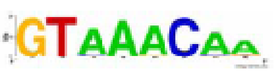	1
GAT1	**GATAA**	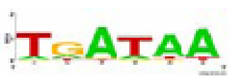	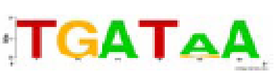	1
GCN4	AR**TGACTCW**	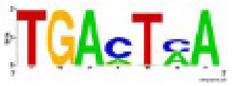	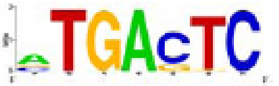	1 shift
HAP4	YCN**NCCAATN**ANM	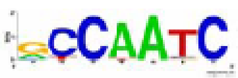	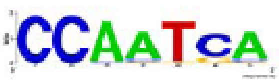	1 shift
INO2	ATTT**CACATGC**	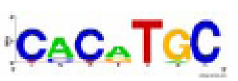	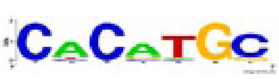	1
INO4	**CATGTGAA**AT	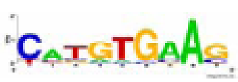	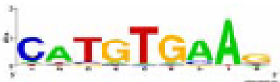	2
LEU3	YG**CCGGTACCGG**YK	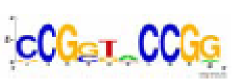	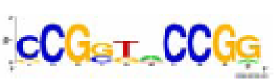	1

MBP1	**ACGCGT**	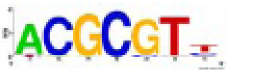	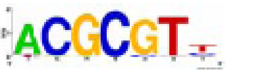	1
MSN2	**MAGGGG**	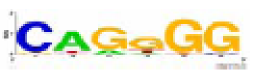	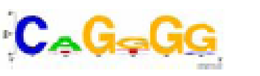	3
NRG1	**CCCT**	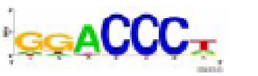	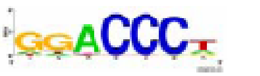	1
PHO2	**ATTA**	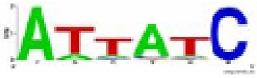	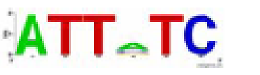	1
PHO4	**CACGTK**NG	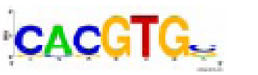	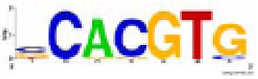	1<br>shift
RCS1	AAMT**GGGTGCA**KT	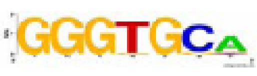	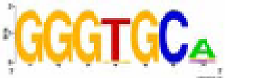	1
RDS1	**KCGGCCG**	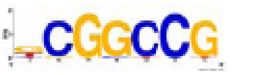	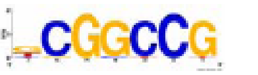	1
STE12	A**TGAAAC**	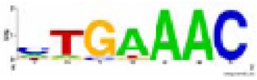	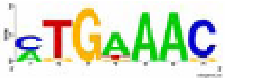	1
SWI4	**CNCGAAA**	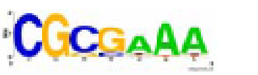	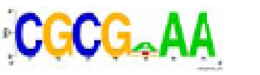	3
TEC1	**CATTC**Y	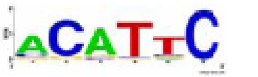	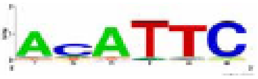	1
TYE7	**CANNTG**	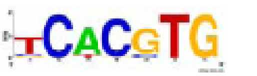	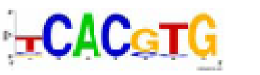	1
UME6	W**GCCGCCG**W	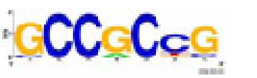	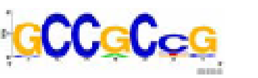	1
YAP1	**TTASTM**A	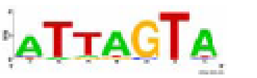	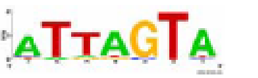	1
YAP7	**TTACTAA**	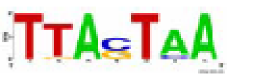	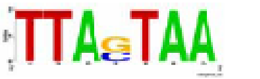	1
HSF1	**TTCTAGAA**NNTTCT	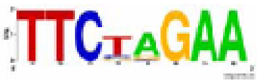	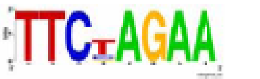	1
RPN4	**GGTGGCAAA**	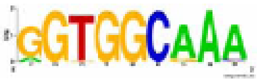	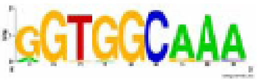	1

ZAP1	**ACC**CTA**AAGG**T	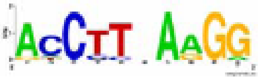	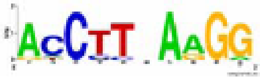	1
RAP1	WRM**ACCCATACA**YY	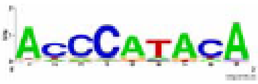	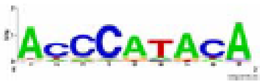	1
MCM1	W**TTCCYAA**WNNGGTAA	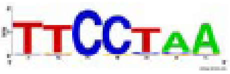	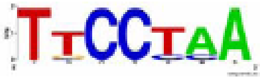	2
